# Essential role of mitogen-activated protein kinases in IL-17A-induced MMP-3 expression in human synovial sarcoma cells

**DOI:** 10.1186/s13104-016-1892-y

**Published:** 2016-02-05

**Authors:** Takuma Sakurai, Daigo Yoshiga, Wataru Ariyoshi, Toshinori Okinaga, Hiroyasu Kiyomiya, Junya Furuta, Izumi Yoshioka, Kazuhiro Tominaga, Tatsuji Nishihara

**Affiliations:** Division of Infections and Molecular Biology, Department of Health Promotion, Kyushu Dental University, 2-6-1 Manazuru, Kokurakita-ku, Kitakyushu, Fukuoka 803-8580 Japan; Division of Oral and Maxillofacial Surgery, Department of Science of Physical Functions, Kyushu Dental University, 2-6-1 Manazuru, Kokurakita-ku, Kitakyushu, Fukuoka 803-8580 Japan; Division of Oral Medicine, Department of Science of Physical Functions, Kyushu Dental University, 2-6-1 Manazuru, Kokurakita-ku, Kitakyushu, Fukuoka 803-8580 Japan

**Keywords:** Interleukin-17, Synovial sarcoma, Matrix metalloproteinase 3, Mitogen-activated protein kinases, Transcription factor AP-1

## Abstract

**Background:**

The tumor cells were needed to rearrange the extracellular matrix (ECM) and reorganize their cytoskeleton to facilitate the cell motility during the tumor invasion. The proinflammatory cytokine interleukin-17A (IL-17A) is reported to up-regulate tumor invasiveness via ECM degradation by matrix metalloproteinases (MMPs). However the precise effects of IL-17A-dependent invasion remain to be characterized. The aim of this study was to elucidate the mechanisms underlying IL-17A-induced MMP-3 expression in the human synovial sarcoma cells HS-SY-II.

**Methods:**

HS-SY-II cells were incubated with IL-17A. In some experiments, the cells were pre-incubated with an anti-IL-17 receptor polyclonal antibody (IL-17R Ab) or inhibitors for signaling cascade prior to addition of IL-17A. The expression of MMP-3 was determined by real-time reverse-transcription polymerase chain reaction (RT-PCR) and western blotting. IL-17R expression in HS-SY-II cells was assessed by immunofluorescence microscopy, while the phosphorylation of signaling molecules was measured by western blotting.

**Results:**

IL-17A increased MMP-3 mRNA and protein expression. HS-SY-II cells express the IL-17R on their surface and blockage of IL-17A-IL-17R binding by IL-17R Ab suppressed IL-17A-mediated induction of MMP-3. IL-17A induced the phosphorylation of three components of the mitogen-activated protein kinase (MAPK) pathway including extracellular signal-regulated kinase 1/2 (ERK1/2), p38 MAPK, and c-Jun NH_2_-terminal kinase (JNK). Pre-treatment of the cells with inhibitors of ERK1/2, p38 MAPK, and JNK attenuated the IL-17A-induced phosphorylation of activator protein-1 (AP-1) subunits and the expression of *MMP*-*3* mRNA.

**Conclusion:**

Our results indicate an essential role for MAPKs in the induction of MMP-3 in synovial sarcoma cells, through AP-1 activation.

## Background

Synovial sarcoma is a clinically aggressive malignant soft tissue tumor and the 5- and 10-year survival rates have been reported as low as 36 and 20 %, respectively [1]. Furthermore, synovial sarcomas have a high rate of local recurrence and metastasis [2]. Therefore, establishment of efficient therapeutic strategies are required to improve the prognosis of synovial sarcoma. Inflammation is a key regulatory process during tumor development. Continuous exposure to inflammatory cytokines is known to cause tumorigenesis [3]. Thus, these cytokines are of interest as therapeutic targets to prevent tumor progression.

Pro-inflammatory cytokine interleukin-17A (IL-17A) is a major member of IL-17 family and is secreted mainly by Th17 cells [4]. The elevated expression of IL-17A has been implicated in the pathogenesis of a wide range of inflammatory, infectious, and autoimmune diseases [5]. In addition, accumulating data showed that the role of IL-17A in cancer initiation, growth, and metastasis was crucial [6–8].

Matrix metalloproteinases (MMPs) degrade a wide range of substrates, including extracellular matrix (ECM) components [9], and numerous studies demonstrated that elevated levels of MMPs are associated with tumor growth, cancer progression, metastasis, and shortened survival in patients [10, 11]. MMP-3 has been recognized as one of the major proteases responsible for ECM turnover and cell–cell interactions, as well as tumor metastasis [12, 13].

The IL-17 family signals via their correspondent receptors activate downstream pathways, including mitogen-activated protein kinases (MAPKs). The MAPK signal transduction pathway, comprising extracellular signal-regulated kinase 1/2 (ERK1/2), p38 MAPK, and c-Jun NH_2_-terminal kinase (JNK), controls gene expression, via the phosphorylation and regulation of transcription factors, co-regulatory proteins, and chromatin.

Previous studies showed that IL-17A induces MMP-1 and MMP-9 via ERK1/2 and p38 MAPK-dependent activation of the transcriptional factors activator protein-1 (AP-1) and nuclear factor-kappa B (NF-κB) [14, 15]. Although both IL-17A and MMP-3 are known to be involved in the initiation and progression of tumor cells, their interaction has yet to be determined. In the present study, we examined the molecular mechanism by which IL-17A-induced MMP-3 expression in synovial sarcoma cells and demonstrated that IL-17 could regulate the expression of MMP-3 through MAPKs-AP-1 activation.

## Methods

### Reagents

Recombinant human IL-17A and anti-JNK monoclonal antibody were purchased from R&D systems (Minneapolis, MN, USA). Anti-phospho-ERK monoclonal, anti-phospho-p38 MAPK monoclonal, anti-phospho-JNK polyclonal, anti-phospho-c-Jun monoclonal, anti-phospho-c-Fos monoclonal, anti-ERK monoclonal, anti-p38 MAPK monoclonal, anti-JNK polyclonal, anti-c-Jun monoclonal, and anti-c-Fos monoclonal antibodies were purchased from Cell Signaling Technology (Beverly, MA, USA). Anti-phospho-IκB-α monoclonal, and anti-IκB-α polyclonal antibodies were purchased from Santa Cruz Biotechnology (Dallas, TX, USA). Anti-MMP-3 polyclonal antibody was purchased from Abcam (Tokyo, Japan). Anti-β-actin monoclonal antibody was obtained from Sigma (St. Louis, MO, USA).

### Cell culture

HS-SY-II human synovial sarcoma cells were purchased from Riken BRC (Ibaraki, Japan). The cells were maintained in Dulbecco’s Modified Eagle Medium (DMEM) supplemented with 10 % fetal bovine serum (FBS), 100 units/ml penicillin G potassium salt, and 140 μg/ml streptomycin sulfate at 37 °C in an atmosphere of 5 % CO_2_.

### Quantitative real-time reverse transcription polymerase chain reaction (real time RT-PCR)

Total RNA from HS-SY-II cells were extracted using an RNeasy Mini Kit (Qiagen, Valencia, CA, USA) according to the manufacturer’s instructions. The RNA was transcribed with ReverTra Ace^®^ quantitative PCR RT Master Mix (Toyobo, Life Science Department, Osaka, Japan) and amplified using a Mastercycler gradient (Eppendorf, Hamburg, Germany). PCR products were detected using FAST SYBR Green Master Mix (Applied Biosystems, Foster City, CA, USA) with the following primer sequences: *GAPDH*, forward 5′-ATG GAA ATC CCA TCA CCA TCT T and reverse 5′-CGC CCC ACT TGA TTT TGG; *MMP*-*3,* forward 5′-TCG TTG CTG CTC ATG AAA TTG and reverse 5′-AGC TTC AGT GTT GGC TGA GTG A. Thermal cycling and fluorescence detection were performed using a StepOne Real-Time PCR System (Applied Biosystems). Relative changes in gene expression were calculated using the comparative CT method. Total cDNA abundance between samples was normalized using primers specific to the *GAPDH* gene.

### Western blotting analysis

Total protein was extracted from the cells using sodium dodecyl sulfate (SDS) lysis buffer (75 mM Tris–HCl containing 2 % SDS and 10 % glycerol, pH 6.8) and protein contents were measured using a DC protein assay kit (Bio-Rad, Hercules, CA, USA). Equivalent amounts (30 μg) of total protein were electrophoresed using e-PAGEL (Atto, Tokyo, Japan), then transferred to polyvinylidene difluoride membranes (Merck Millipore, Billerica, MA, USA). Non-specific binding sites were blocked by immersing the membrane in Blocking one (Nakarai Tesque, Kyoto, Japan) for 1 h at room temperature after which the membranes were treated with the diluted primary antibodies overnight at 4 °C, followed by horseradish-peroxidase-conjugated secondary antibodies (GE Healthcare, Little Chalfont, UK) for 1 h at room temperature. After washing the membranes, chemiluminescence was produced using the ECL western blotting detection reagent or the ECL Prime western blotting detection reagent (both from GE Healthcare UK, Buckinghamshire, UK). Band densities were determined using ChemiDoc™^®^ XRS Plus molecular imager system (Bio-Rad Laboratories). The blots were quantified by measuring the relative band intensity normalized to changes in the β-actin intensity using Image Lab™^®^2.0 software (Bio-Rad Laboratories).

### MMP-3 expression in HS-SY-II cells stimulated with IL-17A

HS-SY-II cells were incubated with IL-17A (10 ng/ml) for 0-24 h. The mRNA level of *MMP*-*3* was measured by real-time RT-PCR. To determine the expression of MMP-3 protein, whole-cell lysates were subjected to SDS-PAGE and western blot analysis, with the blots probed for MMP-3. Equivalent protein aliquots of cell lysates were also analyzed for β-actin.

### Immunofluorescence microscopy for IL-17R

HS-SY-II cells were cultured in 4-well Lab-Tek™^®^ parmanox chamber slides (Nagle Nunc International, Rochester, NY, USA) at a density of 1 × 10^4^ cells/well. The cells were fixed with 4 % paraformaldehyde for 30 min at 4 °C, and quenched with 0.2 M glycine in phosphate-buffered saline (PBS, pH7.2). Specific binding sites were blocked with 1 % bovine serum albumin in PBS for 30 min at room temperature. The cells were then treated overnight at 4 °C with rabbit polyclonal anti-IL-17R (1:100; Santa Cruz Biotechnology), washed three times with PBS, and treated with Alexa Flour^®^ 488 conjugated goat anti-rabbit IgG (1:100; Molecular Probe, Invitrogen, Carlsbad, CA, USA) for 1 h at room temperature. After washing in PBS, the cells were incubated with Alexa Fluor^®^ 568 Phalloidin (1:150; Molecular Probe, Invitrogen) for 15 min at room temperature, followed by the addition of the nuclear staining agent 4′, 6-diamino-2-phenylidole (DAPI). The cells were visualized using a BZ-9000 fluorescence microscope (Keyence, Osaka, Japan). Images were captured digitally in real time and processed using the BZ-II imaging software (Keyence).

### Effect of IL-17R neutralizing antibody on IL-17A-induced MMP-3 expression in HS-SY-II cells

HS-SY-II cells were pre-treated with neutralizing antibody against IL-17R (IL-17R Ab; 0.5-5 μM; Cell Signaling Technology) for 1 h, then incubated with or without IL-17A (10 ng/ml) for 12 h. The mRNA level of *MMP*-*3* was measured by real-time RT-PCR.

### MAPKs phosphorylation in HS-SY-II cells stimulated with IL-17A

HS-SY-II cells were stimulated with IL-17A (10 ng/ml) for 0–300 min. Whole-cell lysates were subjected to SDS-PAGE and western blot analysis, with the blots probed for phosphorylated ERK1/2, p38 MAPK, and JNK. Following visualization, the blots were stripped and re-probed for ERK1/2, p38 MAPK, and JNK. Equivalent protein aliquots of cell lysates were also analyzed for β-actin.

### Effect of MAPKs inhibitors on IL-17A-induced MMP-3 expression in HS-SY-II cells

HS-SY-II cells were pretreated or not with inhibitors of ERK1/2 (U0126, 10 μM; Calbiochem, Darmstadt, Germany), p38 MAPK (SB239063, 10 μM; Calbiochem), and JNK (SP600125, 10 μM; Calbiochem) for 1 h, followed by incubation with 10 ng/ml IL-17A for 12 h. The mRNA level of *MMP*-*3* was measured by real-time RT-PCR.

### c-Fos, c-Jun and IκB-α activation in HS-SY-II cells stimulated with IL-17A

HS-SY-II cells were stimulated with IL-17A (10 ng/ml) for 0–300 min. Whole-cell lysates were subjected to SDS-PAGE and western blot analysis, with the blots probed for phosphorylated c-Fos, c-Jun, and IκB-α. Following visualization, the blots were stripped and re-probed for c-Fos, c-Jun and IkB-α. Equivalent protein aliquots of cell lysates were also analyzed for β-actin.

### Effect of AP-1 inhibitor on IL-17A-induced MMP-3 expression in HS-SY-II cells

HS-SY-II cells were cultured with inhibitor of AP-1 (SR11302, 0.1-1 μM; R&D systems) for 1 h, then incubated in the presence or absence of IL-17A (10 ng/ml) for 12 h. The mRNA level of *MMP*-*3* was measured by real-time RT-PCR.

### Effect of MAPKs inhibitors on IL-17A-induced c-Fos and c-Jun phosphorylation in HS-SY-II cells

HS-SY-II cells were pretreated or not with 10 μM U0126, 10 μM SB239063, and 10 μM SP6000125 for 1 h, followed by incubation with 10 ng/ml IL-17A for 3 h. Whole-cell lysates were subjected to SDS-PAGE and western blot analysis, with the blots probed for phosphorylated c-Fos and c-Jun. Following visualization, the blots were stripped and re-probed for c-Fos and c-Jun. Equivalent protein aliquots of cell lysates were also analyzed for β-actin.

### Statistical analysis

All statistical analyses were carried out using statistical software EZR (Easy R, Saitama Medical center, Saitama, Japan: http://www.jichi.ac.jp/saitama-sct/SaitamaHP.files/statmed.html), which is based on the R and R commander [16]. All data were expressed as the mean ± standard deviation (SD) obtained from three independent experiments and analyzed by a pairwise *t* test with a Holm-Bonferroni correction for multiple comparisons. *P* < 0.01 were considered to be statistically significant.

## Results

### Effect of IL-17A-IL-17R interaction on MMP-3 production by HS-SY-II cells

**Fig. 1:**
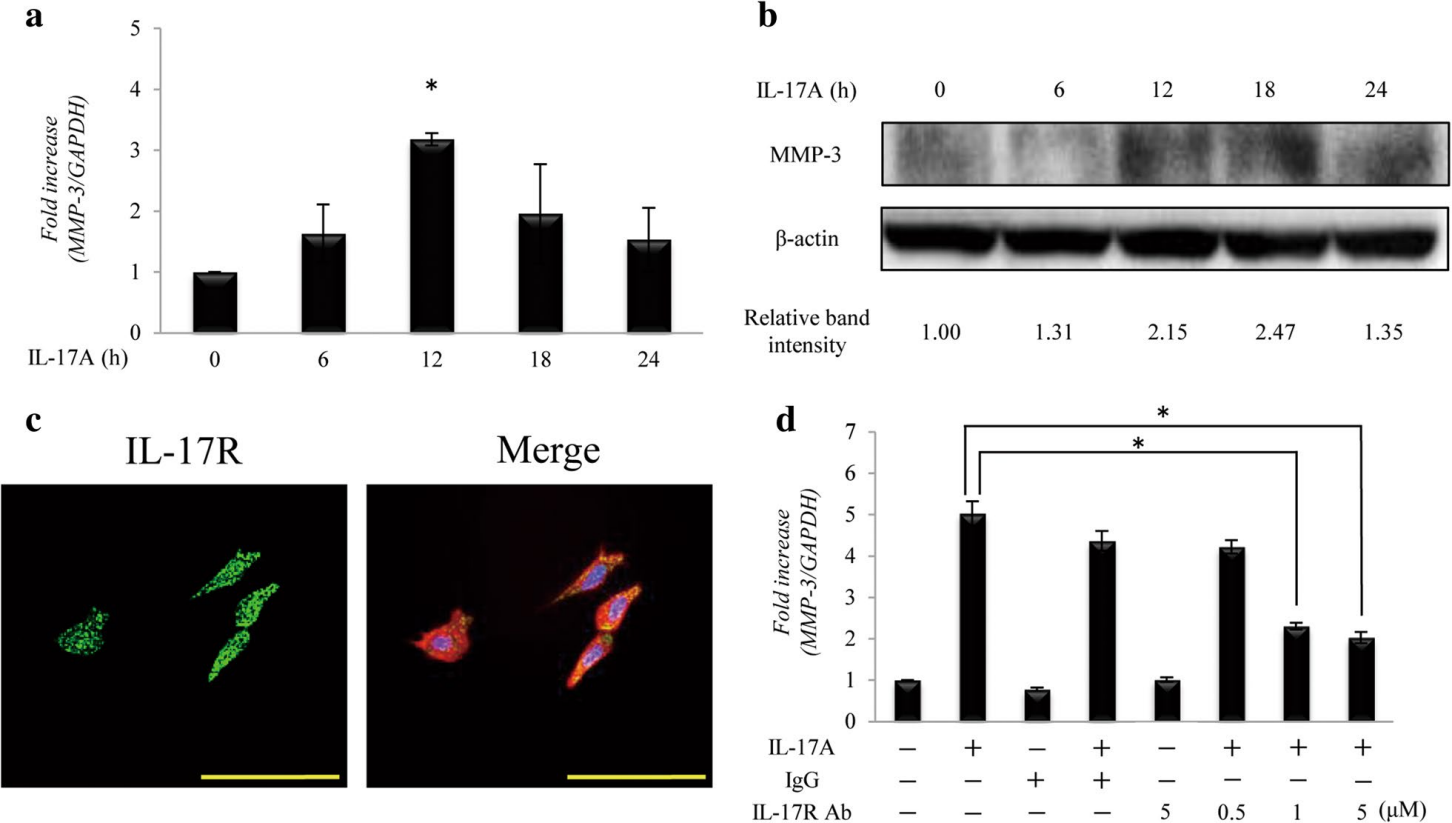
Effect of IL-17A on MMP-3 production by HS-SY-II cells. **a** Representative the mRNA level of *MMP*-*3* in HS-SY-II cells stimulated with IL-17A. Data are expressed as the mean ± SD of triplicate cultures. *Indicates statistical significance of *p* < 0.01. **b** Western blot for MMP-3 in HS-SY-II cells stimulated with IL-17A. **c** Represents immunofluorescent staining for IL-17R in HS-SY-II cells. Merge image is digital three-color overlay of IL-17R (*green*), rhodamine isothiocynate-phalloidin (*red*), and DAPI detection of nuclei (*blue*). The *bars* indicate 100 μm. **d** Representative the mRNA level of *MMP*-*3* in HS-SY-II cells pretreated with IL-17R Ab, followed by incubation with IL-17A. Data are expressed as the mean ± SD of triplicate cultures. *Indicates statistical significance of *p* < 0.01

HS-SY-II cells were incubated in the presence or absence of IL-17A. Figure 1a shows the time-dependent enhancement of *MMP*-*3* mRNA by IL-17A. Maximum enhancement, as determined by real time RT-PCR analysis (3.2-fold increase), occurred after 12 h of stimulation. Western blotting showed that the expression of MMP-3 protein was substantially enhanced in HS-SY-II cells treated for 18 h with IL-17A (Fig. 1b). Immunofluorescence analysis revealed the constitutive cell-surface expression of IL-17R by HS-SY-II cells (Fig. 1c). To further examine the role of IL-17R as an IL-17A receptor in enhancement of MMP-3, HS-SY-II cells were pre-treated with IL-17R neutralizing antibody before the addition of IL-17A. The potent induction of *MMP*-*3* mRNA induced by IL-17A was blocked by IL-17R neutralizing antibody in a dose-dependent manner (Fig. 1d).

### Effect of ERK1/2, p38 MAPK, and JNK on IL-17A-induced MMP-3 expression

**Fig. 2:**
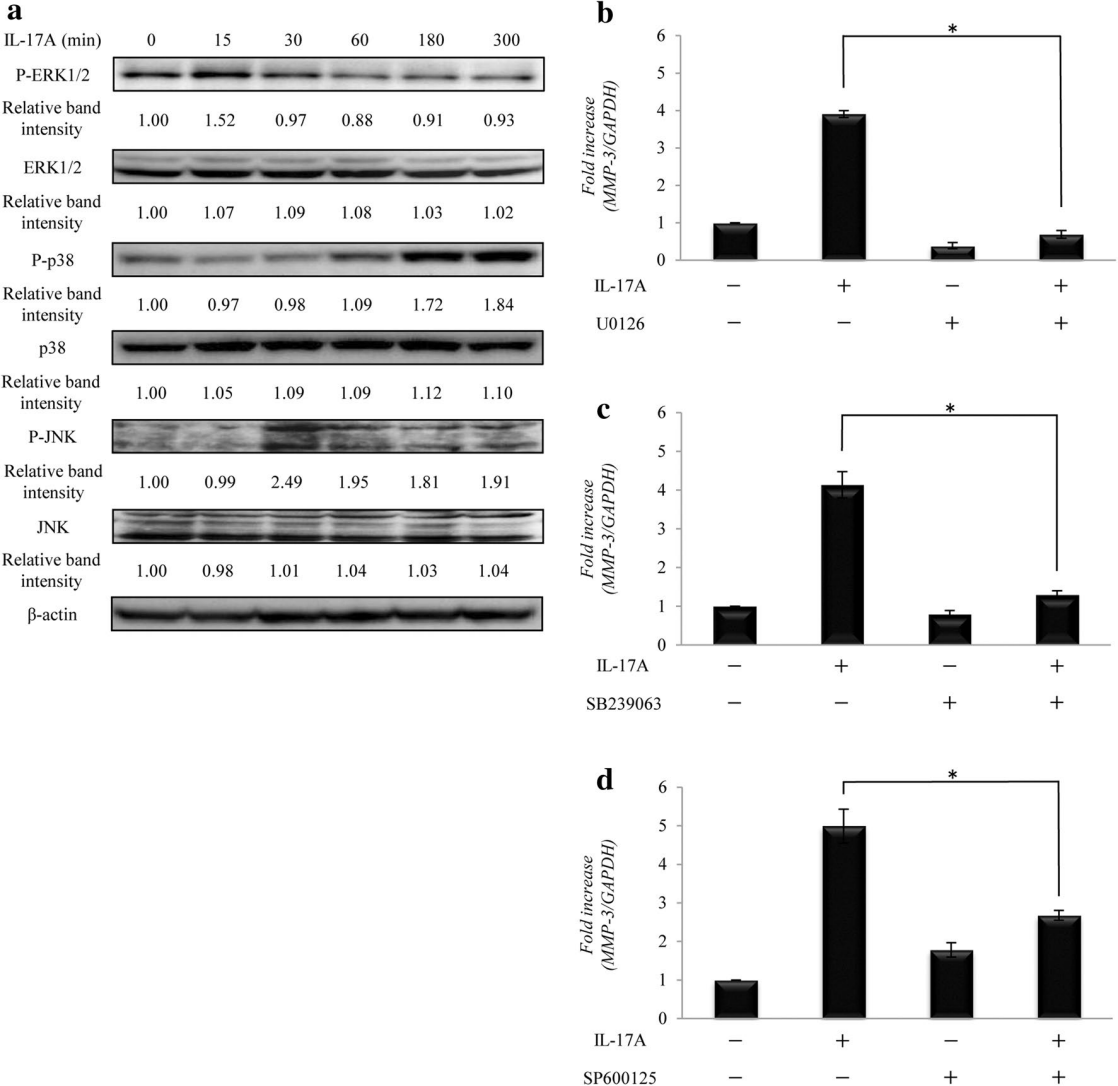
Effects of ERK1/2, p38 MAPK, and JNK on IL-17A-induced MMP-3 expression. **a** Western blot for components of the MAPKs pathway in HS-SY-II cells stimulated with IL-17A. **b**–**d** Representative the mRNA level of *MMP*-*3* in HS-SY-II cells pretreated with U0126 (**b**), SB239063 (**c**), and SP6000125 (**d**), followed by incubation with IL-17A. Data are expressed as the mean ± SD of triplicate cultures. *Indicates statistical significance of *p* < 0.01

Treatment with IL-17A enhanced the phosphorylation of three components of the MAPKs pathway, ERK1/2 (P-ERK1/2), p38 MAPK (P-p38) and JNK (P-JNK), after 15–180 min (Fig. 2a). Thus, we next investigated whether the inhibition of any of these proteins affected the IL-17A-induced expression of MMP-3 in HS-SY-II cells. The cells were pre-treated with specific inhibitors of ERK1/2 (U0126), p38 MAPK (SB239063), and JNK (SP600125), and then stimulated with IL-17A. Real time RT-PCR revealed that the inhibition of ERK1/2 (Fig. 2b), p38 MAPK (Fig. 2c), and JNK (Fig. 2d) abolished the ability of IL-17A to induce *MMP*-*3* mRNA expression.

### Effect of AP-1 activation on IL-17A-induced MMP-3 expression

**Fig. 3:**
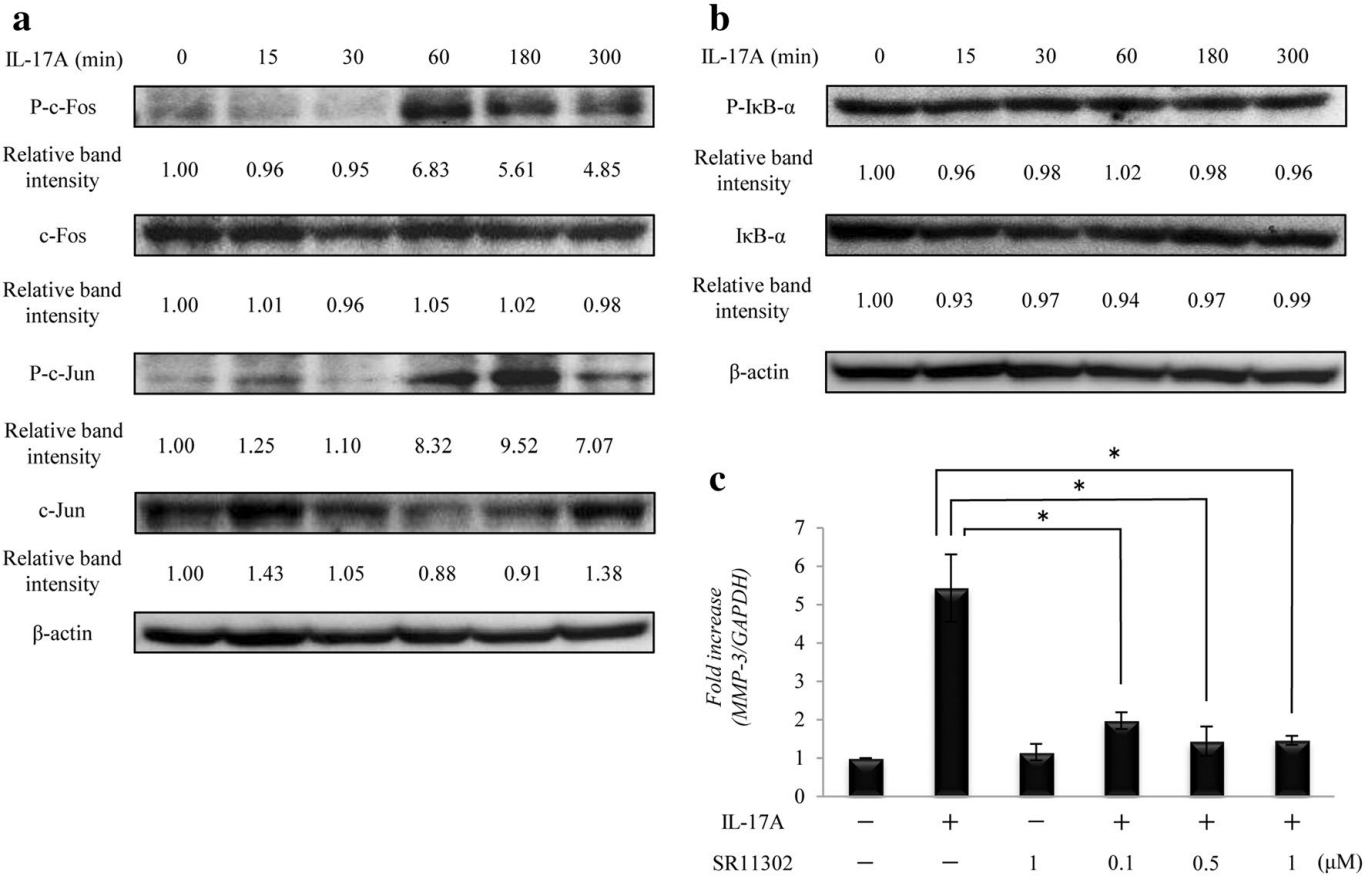
Effect of AP-1 activation on IL-17A-induced MMP-3 expression. **a**, **b** Western blot for components of the AP-1 transcription factor complex (**a**) and IκB-α (**b**) in HS-SY-II cells stimulated with IL-17A. **c** Representative the mRNA level of *MMP*-*3* in HS-SY-II cells pretreated with SR11302, followed by incubation with IL-17A. Data are expressed as the mean ± SD of triplicate cultures. *Indicates statistical significance of *p* < 0.01

IL-17A stimulation resulted in the phosphorylation of c-Fos (P–c-Fos) and c-Jun (P–c-Jun), the major components of the AP-1 transcription factor complex, in a time-dependent manner. Activation occurred within 180 min (Fig. 3a), whereas IL-17A had no effect on NF-κB activation, as evidenced by phosphorylation (P-IκB-α) or degradation of IκB-α (Fig. 3b). To determine the role of AP-1 activity in IL-17A-induced expression of MMP-3, HS-SY-II cells were pre-treated with AP-1 blocking synthetic peptide (SR11302) prior to their stimulation with IL-17A. Real time RT-PCR revealed that SR11302 pre-treatment effectively blocked IL-17A-induced *MMP*-*3* mRNA expression in a dose-dependent manner (Fig. 3c).

### Effect of ERK1/2, p38 MAPK, and JNK inhibitors on AP-1 activation mediated by IL-17A

**Fig. 4:**
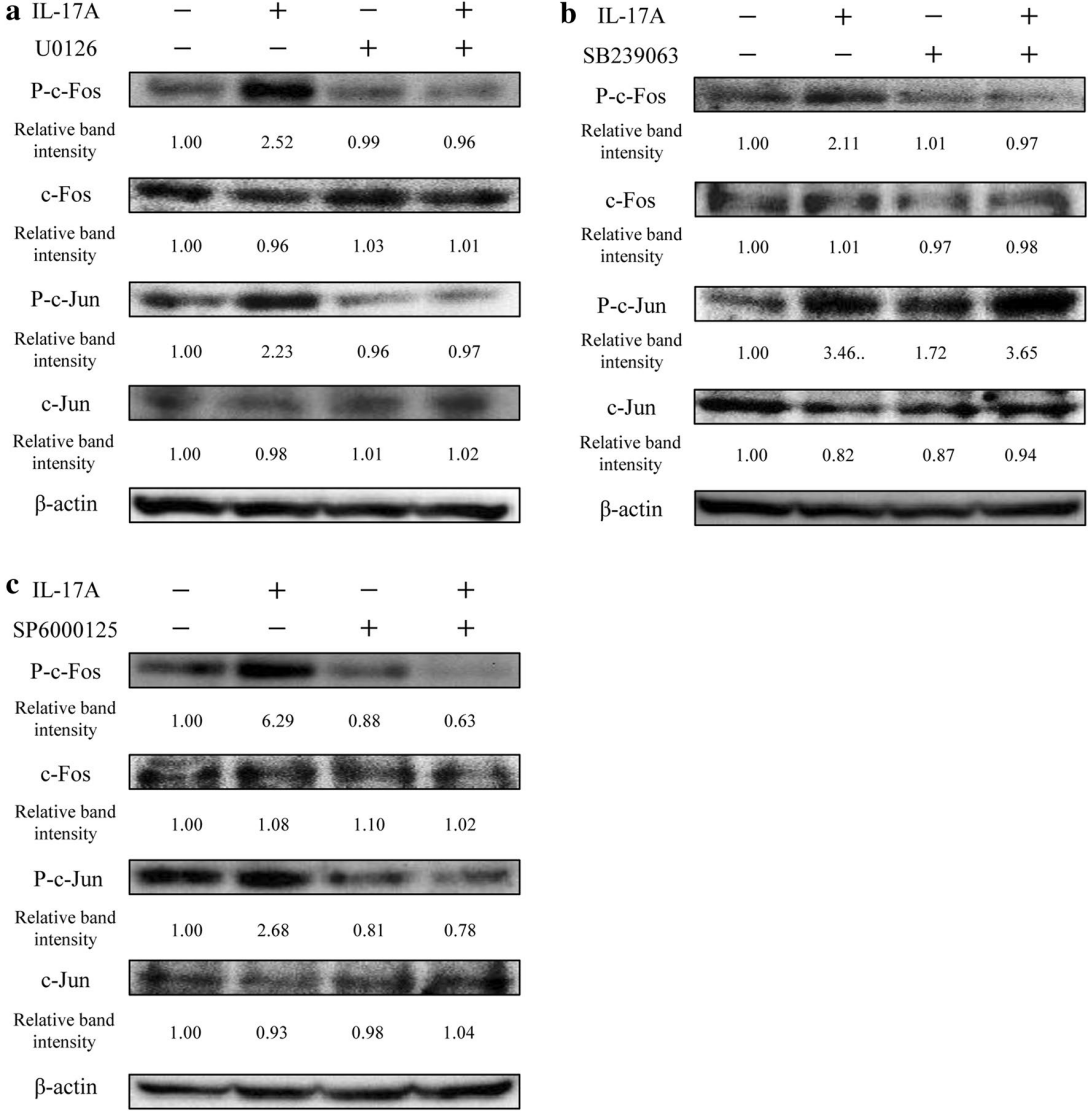
Effects of ERK1/2, p38 MAPK and JNK inhibitors on AP-1 activation mediated by IL-17A. **a**–**c**: Western blot for components of the AP-1 transcription factor complex in HS-SY-II cells pretreated with U0126 (**a**), SB239063 (**b**), and SP6000125 (**c**), followed by incubation with IL-17A

Since the activation of MAPKs and AP-1 was necessary for IL-17A-induced MMP-3 expression in HS-SY-II cells, we asked whether the phosphorylation of ERK1/2, p38 MAPK, and JNK was associated with AP-1 activation. HS-SY-II cells were pre-treated with the inhibitors U0126 (ERK1/2), SB239063 (p38 MAPK), and SP600125 (JNK) and then stimulated with IL-17A. Western blotting revealed that U0126 and SP600125 attenuated the IL-17A-induced phosphorylation of c-Fos and c-Jun (Fig. 4a, c) whereas SB239063 suppressed the IL-17A-stimulated phosphorylation of c-Fos but not c-Jun (Fig. 4b).

## Discussion

Since previous studies have shown that MMP-3 is capable of stimulating spontaneous tumor development in mammary gland and lung [17–19], knowledge of MMP-3 regulation is of importance for developing therapeutic strategies. On the other hand, the link between inflammation and carcinogenesis is well known that extracellular factors, including cytokines and growth factors, have been implicated in the regulation in different types of tumor cells [20, 21]. Previous study reported that IL-17A and IL-17R interaction enhanced the metastasis of osteosarcoma cells via the expression of VEGF, MMP-9 and CXCR4 [22]. In the present study, we have explored the role of IL-17A in the MMP-3 expression of synovial sarcoma cell lines, HS-SY-II.

The results of this study indicated that MMP-3 transcription and protein synthesis are stimulated in HS-SY-II cells in response to IL-17A. The binding of IL-17A to its receptor is thought to mediate the biological effects of IL-17A [23]. IL-17R is an ubiquitous transmembrane glycoprotein which is expressed by several types of cells including tumor cells [24, 25]. In this study, we first confirmed the constitutive cell-surface expression of IL-17R by HS-SY-II cells. We also demonstrated that IL-17R neutralizing antibody remarkably inhibited the effect of IL-17A on MMP-3 expression. These findings suggest that the stimulatory effect of IL-17A on MMP-3 expression is due to its interaction with IL-17R.

After the binding of IL-17A to its receptor, Act1 associates with IL-17R, followed by the activation of downstream signaling molecules including MAPKs [26], important mediators of a variety of physiopathological cellular processes, including cell death, cell survival, proliferation, and migration [27]. In the present study, we found that the ERK1/2, p38 MAPK and JNK pathways were rapidly and transiently activated in cells treated with IL-17A. Moreover, chemical inhibitors of ERK1/2, p38 MAPK, and JNK down-regulated the IL-17A-induced stimulation of *MMP*-*3* mRNA expression. Therefore, we speculated that induction of MMP-3 expression in IL-17A-treated HS-SY-II cells may occur because of activation of MAPKs.

MMP promoters harbor *cis*-elements that allow the regulation of MMP gene expression by a diverse set of trans-activators, including AP-1 [28]. Various stimuli lead to the activation of c-Fos and c-Jun products, which heterodimerize and bind to AP-1 sites within *MMP*-*3* gene promoters [29]. Previous studies reported that the phosphorylation of both c-Fos and c-Jun was involved in AP-1 activation [30] and that the translocation of phosphorylated c-Jun from the cytoplasm to the nucleus activated AP-1 [15, 31]. Western blotting revealed that the phosphorylation of c-Fos and c-Jun protein was enhanced by IL-17A. The importance of AP-1 activation in IL-17A-stimulated MMP-3 expression was demonstrated clearly by the dramatic decrease in *MMP*-*3* gene expression induced by SR11302.

It is also well known that AP-1 transactivation, by increasing the abundance of AP-1 components and/or altering the phosphorylation of its subunits c-Fos and c-Jun, is regulated by the MAPKs pathway [32, 33]. We investigated the relationship between MAPKs and AP-1 in HS-SY-II cells stimulated with IL-17A and found that ERK1/2, p38 MAPK, and JNK, all of which are activated by IL-17A, were required for IL-17A-induced c-Fos phosphorylation. In addition, ERK1/2 and JNK activation were shown to be involved in IL-17A-induced c-Jun phosphorylation. These results provide evidence of an important link between MAPKs activation and AP-1 in HS-SY-II cells stimulated with IL-17A, and strongly support our speculation that MAPKs activation regulates MMP-3 expression. To further determine the interaction between MAPKs and AP-1 in HS-SY-II cells, silencing of MAPKs expression by siRNA or shRNA will be needed.

It has been reported that the chromosomal translocation t (X; 18) that produces the chimeric gene *SYT*-*SSX* at high frequency in synovial sarcomas [34–38] and that the expression of *SYT*-*SSX* is considered to play a central role in tumorigenesis [39]. HS-SYII cells genetically possess the *SS18*-*SSX1*, whereas other human sarcoma cell lines, SYO-1 and Fuji cells have another type of chimeric gene *SS18*-*SSX2*. Further studies are needed to examine the effect of IL-17A/IL-17R interaction on viability, migration and invasion of other human sarcoma cells lines, as well as of primary synovial sarcoma tissue.

These findings might provide mechanistic explanation for the pathogenesis in synovial sarcoma, and suggested that targeting IL-17A/IL-17R pathway was a novel promising strategy to treat patients with synovial sarcoma.

## Conclusion

Our preliminary findings provide a valuable insight how IL-17A may contribute to the pathogenesis of synovial sarcoma by stimulating MMP-3 expression. We showed that the interaction of IL-17A with its receptor, IL-17R, stimulates ERK1/2, p38 MAPK, and JNK activation, which in turn initiates the activation of AP-1, followed by an increase in MMP-3 expression.

## Abbreviations

IL-17A: interleukin-17A
MMPs: matrix metalloproteinases
ECM: extracellular matrix
MAPK: mitogen-activated protein kinase
ERK1/2: extracellular signal-regulated kinase ½
JNK: c-Jun NH_2_-terminal kinase
AP-1: activator protein-1
NF-κB: nuclear factor-kappa B
IL-17R: IL-17 receptor
DMEM: Dulbecco’s Modified Eagle Medium
FBS: fetal bovine serum
RT-PCR: reverse transcription polymerase chain reaction
SDS: sodium dodecyl sulfate
PBS: phosphate-buffered saline
DAPI: 4′, 6-diamino-2-phenylidole
SD: standard deviation
